# Feasibility, acceptability and lessons learned from an infant feeding intervention trial among women living with HIV in western Kenya

**DOI:** 10.1186/s12889-023-16794-2

**Published:** 2023-10-05

**Authors:** Ann E. Maltby, Belinda C. Odhiambo, Maureen Nyaura, Rosemary Shikari, Emily L. Tuthill

**Affiliations:** 1 Department of Community Health Systems, School of Nursing, University of California, San Francisco, CA USA; 2 Ambercare Medical Centre, Kisumu, Kenya

**Keywords:** PMTCT, WLWH, Breastfeeding, Exclusive breastfeeding, Pregnancy, Lactation support, Lactation specialist

## Abstract

**Background:**

The World Health Organization recommends mothers breastfeed exclusively for the first six months of their infant’s life. However, women living with HIV in low resource settings face many barriers to recommended infant feeding practices such as fear of HIV transmission and perceived milk insufficiency. Moreover, current support for breastfeeding in this context is often insufficient to overcome these barriers. To support women’s infant feeding experience, we tested a personalized infant feeding support program among perinatal women living with HIV in Kenya.

**Methods:**

*Supporting Healthy Mothers* is a theory and evidence-based multilevel intervention designed to address the mental health burden associated with financial and food insecurity and provide personalized support for optimal infant feeding postpartum. As part of the *Supporting Healthy Mothers* intervention feasibility trial, between February 23, 2022 and November 9, 2022, twenty mothers received five personalized infant feeding support sessions delivered by a local professional lactation specialist from pregnancy until three months postpartum. Through detailed observations of these sessions, clinical notes and repeated team discussions, we aimed to describe and provide a limited evaluation of these sessions. We identified the strengths and limitations of the lactation support sessions as well as areas for future development.

**Results:**

Participation in the sessions was high and at three months postpartum all participants reported exclusive breastfeeding as recommended despite experiencing a myriad of challenges. Having face-to-face and frequent early postpartum sessions, being available to field participant concerns between sessions and measuring infant weights at each session were key strengths. Continuing sessions beyond three months postpartum and incorporating family planning and general maternal health counseling topics would enhance these supportive sessions.

**Conclusions:**

The personalized professional infant feeding support sessions were highly acceptable and feasible to implement. In-person sessions, in a clinic setting provided opportunities to evaluate and adjust breastfeeding technique and led to successful exclusive breastfeeding practice. Future interventions should consider integrating with other perinatal care services and offering support on demand and immediately postpartum.

**Trial registration:**

*Supporting Healthy Mothers* was registered with ClinicalTrials.gov Protocol Registration and Results System, posted on February 2, 2022. Identifiers: NCT05219552 Unique Protocol ID: K23MH116807.

## Background

Breastfeeding exclusively for the first six months of an infant’s life followed by safe and adequate complementary feeding in addition to continued breastfeeding for 24 months or beyond is the recommended practice for all women [1]. However, the practice of breastfeeding requires time, knowledge, skill, commitment and resilience. That women are willing, capable, and have what is required to breastfeed their infants, especially exclusively, should not be presumed.

In Kenya where 890,000 women are living with HIV, the Ministry of Health has widely promoted optimal care for HIV exposed infants including the World Health Organization’s (WHO) recommendation to exclusively breastfeed for the first six months, lifelong antiretroviral therapy (ART) for women, and HIV prophylaxis medications for breastfeeding infants and children [2–7]. Yet, women living with HIV (WLWH) in this setting face many barriers to exclusive breastfeeding such as financial insecurity and the need to return to work, [8] a lack of resources to pump or store milk, fear of HIV transmission, [9] stigma, [10–12] perceived milk insufficiency, [13, 14] a lack of knowledge about infant satiety cues and nutritional requirements, [8] cultural norms such as giving water or food to babies before six months, [15] and pressure from neighbors or family [12].

Despite evidence that counseling from healthcare workers can increase rates of exclusive breastfeeding [8, 16], support from healthcare workers is not always enough to overcome barriers [12, 17]. Moreover, strong messaging from providers can hinder conversations with mothers about the pros and cons of various infant feeding choices [13, 18, 19]. Other challenges with current support for infant feeding include inconsistent, inaccurate or biased messaging, as well as insufficient training, knowledge, assessment skills, personnel and/or educational materials [12, 20–23]. Indeed, a dearth of resources and time constraints imposed by heavy workloads for healthcare providers in this setting has left a gap in support for breastfeeding initiation, technique and timing [23, 24]. Furthermore, support from certified lactation specialists is non-existent in public healthcare facilities in Kenya. Yet, the expectation to exclusively breastfeed remains. Thus, there is a need for a greater number of skilled and knowledgeable providers to engage women in conversations about the risks and benefits associated with infant feeding options, the barriers they face to optimal infant feeding (namely breastfeeding), and practical ways to overcome challenges.

To address this need, we developed *Supporting Healthy Mothers*, a theory and evidence-based multilevel intervention to support pregnant and postpartum (perinatal) WLWH. *Supporting Healthy Mothers* was specifically designed to address the mental health burden associated with financial and food insecurity and provide personalized support for optimal infant feeding postpartum. What follows is an overview of the infant feeding support component of *Supporting Healthy Mothers*. Drawing from clinical notes, detailed observations and team discussions, we describe the infant feeding support activities carried out and give an overview of the feasibility, acceptability, strengths, limitations and recommendations for future development.

## Methods

### Supporting Healthy Mothers intervention 

The *Supporting Healthy Mothers* intervention had two components: (1) Personalized infant feeding support, from pregnancy through three months postpartum, provided by a local professional lactation specialist; and (2) Unconditional cash transfers distributed to participants in ten monthly installments starting in the third trimester of pregnancy to approximately six months postpartum (see Table 1). At baseline, and at regular intervals, questionnaire data were collected from participants on a variety of topics including sociodemographic information, breastfeeding practices, infant feeding intentions, health status, food security and social support. These data were used to personalize the breastfeeding sessions, and were analyzed in relationship to data collected at a control site. The results of these analyses and a more detailed description of the methods used for the *Supporting Healthy Mothers* feasibility trial are presented in a publication currently under review. In what follows, we will focus only on the personalized infant feeding support sessions and the qualitative observational data we collected during the sessions.

Ethical Approval: Written informed consent was obtained for all participants included in the study. Ethical approval was obtained from Kenya Medical Research Institute Scientific and Ethics Review Unit and the University of California San Francisco.

**Table 1:** Supporting Healthy Mothers Activities

Timing	Activities	
20-35-weeks pregnant	Recruitment, consent, unconditional cash transfer (UCT)** paperwork, baseline questionnaire and scales—baseline questionnaire gets sent to LS so she can review it. **Collected from medical records**: viral load and hemoglobin	
28-36-weeks pregnant	Prenatal breastfeeding session with Lactation Specialist (LS) (#1)	
30-weeks pregnancy	*	UCT #1
34-weeks	*	UCT #2
38-weeks	*	UCT #3
Birth	**Collected from medical records**: birth weight, length and # weeks gestation at birth.	
2-weeks postpartum	Breastfeeding session with LS (#2); focused follow-up questionnaire, infant weight obtained by our team.	UCT #4
4-weeks postpartum	Breastfeeding session with LS (#3); 4-week follow-up questionnaire, infant weight obtained by our team.	
6-weeks postpartum	Breastfeeding session with LS (#4); 6-week follow-up questionnaire, infant weight obtained by our team. **Collected from medical records**: Infant HIV test results, weight and length.	UCT #5
10-weeks postpartum	*	UCT #6
12-weeks (3-months) postpartum	Final breastfeeding session with LS (#5); 3-month follow-up questionnaire, infant weight obtained by our team.	
14-weeks postpartum	*	UCT #7
20-weeks postpartum	*	UCT #8
24-weeks (6-months) postpartum	6-month follow-up questionnaire and infant weight. **Collected from medical records**: Infant HIV test results, weight, length, vaccination status, maternal viral load and hemoglobin.	UCT #9
28 weeks postpartum	Final exit interview	UCT#10 (Final payment)

* During any months when no research encounter occurred with participants, the Research Coordinator conducted and documented follow-up phone calls to touch base with participants and confirm the next research encounter. She additionally confirmed that the monthly cash transfer was received by the participant and that there were no adverse events related to participation

**UCTs were distributed to participants regardless of whether or not they participated in the lactation support sessions or other study activities

### Infant feeding support component of Supporting Healthy Mothers

Using findings from a longitudinal qualitative exploration of women’s perinatal experiences in this setting, [25] current literature and the theoretical framework of Transitions Theory, [26, 27] we collaborated closely with the Maternal Child Health clinic staff at a sub-county hospital in Kisumu, Kenya and a locally trained, experienced, certified doula and Lactation Specialist to develop the infant feeding support component of the intervention. We developed a series of culturally sensitive, interactive, infant feeding support sessions to be delivered to participants from pregnancy up to three months postpartum.

Specific content and educational materials for the *Supporting Healthy Mothers* curriculum were developed using the Infant and Young Child Feeding Counselling: An Integrated Course, Trainer’s guide, second edition created and made available by the WHO [28]. In addition, we utilized Maternal Infant and Young Child Nutrition national counseling cards made available by the Republic of Kenya Ministry of Health, the United Nations Children’s Fund (UNICEF), Action Against Hunger, and Feed the Children [29]. The national counseling cards are based on the 2013 Kenyan Policy Guidelines on Maternal, Infant and Young Child Nutrition [2]. See Tables 2, 3 and 4 for an outline of the curriculum

**Table 2:** Prenatal Breastfeeding Support Session

Activity	Key topics
Assess knowledge and experience related to breastfeeding	• Current infant feeding recommendations (highlighting changes from previous)
Facilitate discussions on lactation and breastfeeding	• Ensure the important benefits of exclusive breastfeeding for the first six months after birth are understood, benefits include: o Health benefits to the baby o Health benefits to the mothers o Economic benefits o Perfect nutrition- Breastmilk (unlike porridge) is a complete food and contains everything a baby needs for the first 6months of life. • How breastfeeding works o Breastmilk supply and demand cycle o How to recognize effective milk exchange o The size of the baby’s stomach during the first weeks postpartum o The change in the texture and composition of breastmilk during the first week o The change in breastmilk during one feeding (foremilk/ hindmilk)
Provide practical information about the timing and frequency of breastfeeding during the immediate postpartum period	• On demand feeding • Babies should not go more than 2 hours without feeding (also during the night) • When infants cluster feed and might seem to be feeding constantly and not sleeping this does not indicate a problem, but can be a growth spurt. • Empower moms to be comfortable with their baby’s unique feeding habits—some babies tank up eating a lot all at once, while others keep “snacking” as long as they are awake. • Moms should feed fully on one side before switching to the other side so babies receive both foremilk and hindmilk with each feeding. • When and how to express and store breast milk and prepare breastmilk, if applicable
Provide practical information about infant care for breastfeeding mothers	• Changes in the baby’s stool during the first days/ week postpartum • Signs that a baby is hungry/ full • Signs that a baby is getting enough milk • Infant medication administration • How to burp the baby to reduce gas pains
Provide practical information about self-care for breastfeeding moms	• Mother’s diet and hydration • Mother’s adherence to ART • Role that partners and other supporters play
Identify any barriers to breastfeeding or exclusive breastfeeding including what factors affect a woman’s choice to breastfeed and discuss strategies for overcoming these barriers	Common barriers include: • Previous negative experiences withbreastfeeding • Unsupportive family or friends • Unfounded beliefs: (i.e., breastmilk is not enough for baby boys) • Fear of HIV transmission • Breast health problems • Lack of food and water to support sufficient breastmilk production
Using the breast model and demonstration dolls, demonstrate infant positioning and attachment of the baby onto the breast	• Baby’s tummy to mom’s tummy and baby’s head and neck in alignment with baby’s body • What a deep latch looks like o position of the baby’s lips and tongue o most of the areola covered by the baby’s mouth • What a deep latch feels like o Not painful
Address any additional questions or concerns	• As determined by the participant

**Table 3:** 2, 4, and 6-week Breastfeeding Support Sessions

Activity	Key topics
Observe a feed/ assess mother and infant	• General health of the mother and her baby • Signs of bonding between mother and baby • Position and latch • Effective milk exchange • Breast health
Facilitate a discussion of the mother’s current breastfeeding practice	• Frequency and duration of feeding • Diaper changes (frequency and contents) • Burping and “bicycle riding” to reduce gas pain • Reassess knowledge and opinions about best feeding practices as needed/ • Identify and address difficulties and barriers to continued breastfeeding.
Assess sufficiency of milk supply	• Infant’s weight • Breast assessment • # of wet/ soiled diapers • Observations of milk exchange during feeding.
Assess adherence	• Maternal adherence • Infant adherence • Identify and address difficulties and barriers
Set measurable and attainable goals	• Related to breastfeeding practice • Related to infant care • Related to self-care • Related to adherence
Provide treatment and make recommendations and referrals as applicable	• For example: o Express milk from engorged breast. o Warm/ cold compresses o Recommend and assist with changes to latch or infant’s position o Refer to primary care for treatment of thrush or mastitis
Congratulate mother and provide reassurance and emotional support	• Acknowledge the hard work the mother is doing • Point out the progress being made • Reassure mother of adequacy of milk supply (as applicable) • Reinforce mother’s confidence • Congratulate her success related to breastfeeding, adherence and demonstrated knowledge
Address any additional questions or concerns	• As determined by the participant

**Table 4:** 3-month Breastfeeding Support Session

Activity	Key topics
Review of the mother’s infant feeding practice since the six-week session	• Breastfeeding exclusively, why and why not? • Challenges or barriers • Infant growth • Congratulate and reassure
Provide the mother with strategies to overcome expected challenges from 3–6 months postpartum	Common challenges during this time include: • Increased pressure to introduce complementary foods or stop breastfeeding • Increased difficulties with medication administration • Baby’s growth spurts/ cluster feeding • Baby more easily distracted during breastfeeding—a normal part of development • Teething
Provide practical information about complementary feeding.	• Explore the mother’s current knowledge and past experiences • Debunk myths and misconceptions • Identify and address potential challenges • Discuss: o How to assess baby’s readiness o When and why complementary feeding is needed o Recommended foods (types and amounts) o Recommended amount of water o Frequency of feeding o Positioning during feeding o Responsive feeding techniques
Demonstrate preparation of a baby’s first foods	• Thickness and amount for first feeds • Temperature
Provide practical information about stopping breastfeeding.	• Current recommendations (24-months or beyond) • Considerations for deciding when to stop • How to gradually go about it • When infant HIV prophylaxis can be stopped
Address any additional questions or concerns	• As determined by the participant.

The infant feeding support sessions were carried out between February 23, 2022 and November 9, 2022 in a private office within a sub-county hospital in Kisumu, Kenya. Prior to the first supportive session, the Research Coordinator provided the Lactation Specialist with the relevant information from the participant’s baseline survey so the Lactation Specialist could personalize the session according to the mother’s knowledge, experience, level of confidence and need for, as well as openness to, support. At each visit, participants met with the Research Coordinator who weighed their infant (at postpartum visits) and administered the corresponding electronic questionnaires including a variety of questions to assess infant feeding behaviors (including exclusive breastfeeding). Then, the Research Coordinator introduced the Lactation Specialist, and remained in the room to quietly observe while the Lactation Specialist conducted the infant feeding support sessions. Participants were provided with refreshments during the visit and reimbursed 800 Kenyan Shillings for the cost of their transportation to the clinic.

The first breastfeeding support session occurred during the participant’s third trimester of pregnancy (see Table 2 for the activities and topics covered during this session). After the initial visit, the Lactation Specialist met with women at two, four and six weeks postpartum, where she observed the mothers feeding their babies and assessed their progress (see Table 3 for an overview of the content delivered during these sessions). At the final 3-month postpartum visit, the Lactation Specialist assessed each mother’s progress and prepared them for complementary feeding (recommended to begin after babies reach six-months-old). See Table 4 for an overview of the content delivered during the 3-month session.

### Documentation of the infant feeding support sessions

After each session, the Lactation Specialist wrote or dictated a summary of her encounter with participants including her focused assessment of the participant and her infant, any problems or challenges the participant was facing and the interventions or recommendations she provided. The Research Coordinator, experienced in qualitative methods, also took detailed notes during the sessions. She specifically noted the participant’s level of engagement, body language and facial expressions. She captured the participant’s questions and concerns, how they were addressed by the Lactation Specialist and how the information or support was received by the participant. She then wrote a detailed summary of her observations after each session.

Exit interviews with participants evaluating all components of the *Supporting Healthy Mothers* intervention were conducted at around seven months postpartum. Data collection procedures, analysis and results from these interviews will be presented in a separate publication currently under review.

### Analysis

The aim of our analysis was to assess feasibility—to what extent we executed the infant feeding support sessions according to our plan. We considered whether or not the Lactation Specialist delivered the developed curriculum as planned and if changes to our intervention design were required. We also assessed acceptability—whether or not the sessions were relevant to women (addressed women’s infant feeding problems and concerns) and to what extent women attended the sessions.

Our analysis team consisted of a US-based Primary Investigator, two Research Coordinators based in Kenya, and one Research Coordinator based in Denmark. Collaborating closely, our team utilized the long table approach [30] to collectively analyze: the clinical notes from the Lactation Specialist, the detailed observations of the Research Coordinator, and the log of phone calls fielded by the Research Coordinator between infant feeding support sessions. Specifically, we divided up a hard copy of these data among our four team members. Then, during face-to-face meetings over the course of one week, we carefully reviewed the data to identify all of the excerpts that mentioned women’s infant feeding concerns or challenges and any descriptions of the support provided by the Lactation Specialist to address the concern or challenge (treatment, information, emotional support, reassurance, etc.). We cut up the documents into excerpts labeled with the unique participant identification numbers and organized by timepoint. We then grouped the excerpts according to similar concerns or challenges at each timepoint and summarized our findings for each timepoint.

Ultimately, we reviewed our findings to identify major concerns or challenges that were addressed as related to the planned curriculum. Throughout the course of the analysis, we also reviewed and discussed appointment attendance, participant retention and any other lessons learned.

## Results

### Participants

Twenty women, from the Luo [15] and Luhya [5] ethnic groups ranging in age from 20 to 43-years-old, participated in the infant feeding support component of *Supporting Healthy Mothers*. The majority of women had a primary school education or higher. At baseline, around half of the women lived in rural Kisumu and half within the city, with 11/20 women living in informal housing and an average travel time to the clinic for the group of around 40 min. Fifteen women lived together with the father of their baby, and 5/20 were in polygamous marriages. Sixteen women had unplanned pregnancies and just 4/20 were pregnant for the first time. Five women were diagnosed with HIV within the last 2 years. Most women (17/20) had disclosed their HIV status to their current partner and 100% were engaged in HIV care and reported taking ART at the time they were enrolled in the intervention. Most women (19/20) planned to exclusively breastfeed for some period of time after birth, with only one woman not planning to breastfeed. Survey data also showed that only six women planned to breastfeed beyond 12 months and just three women planned to breastfeed for the recommended 24 months.

### Observations from the infant feeding support sessions

We planned for the Lactation Specialist to meet with 20 women for 5 sessions each from pregnancy through 12 weeks postpartum for a total of 100 infant feeding support sessions. Participants attended 94 of 100 sessions (2 sessions were missed at 2 weeks postpartum and 4 sessions were missed at 4 weeks postpartum). Thus, 94% of sessions were attended as planned, and no participants were lost to follow-up.

### Prenatal: 1st breastfeeding support session during third trimester

During the prenatal session, the Lactation Specialist and Research Coordinator noted a number of fears/worries women were facing: fear of not producing enough breastmilk, fear of pain during breastfeeding, fear of transmitting HIV to their baby, worry that their baby will suffer from colic and worry that others will discover their HIV status if they reveal they are practicing exclusive breastfeeding (exclusive breastfeeding is commonly associated with WLHW in Kenya) [10, 11]. To address these fears, the Lactation Specialist assured mothers that their bodies were capable of producing enough breastmilk and explained the demand and supply concept of breastmilk production. She further reinforced knowledge on the Prevention of Mother to Child Transmission (PMTCT), encouraging mothers to adhere to ART, give birth at the hospital and exclusively breastfeed. For the participant who initially reported she was not planning to breastfeed, support from the Lactation Specialist allowed her to overcome concerns about HIV transmission, and make an informed decision to give exclusive breastfeeding a try.

Women also came to the first session with a number of myths and misconceptions about breastfeeding. Among the most common myths was the idea that breastmilk is not enough for baby boys. Many women (10/20) also believed that babies have “plastic teeth” in their gums that should be removed by a traditional healer (an unsafe traditional practice in this region that involves cutting an infant’s gums and/or the removal of an infant’s primary teeth buds) [31]. The Lactation Specialist provided information to dispel these myths. For example, she explained that, regardless of gender, when a baby is allowed to breastfeed on demand, the baby will stimulate the breast to produce sufficient milk according to that baby’s needs. She also encouraged mothers to come to her first should they suspect their infant has “plastic teeth”.

At the first session, the Lactation Specialist also took time to answer women’s questions, the most common ones being about why one should exclusively breastfeed and what would be the consequence of introducing food, milk or water to their baby before their baby reaches 6-months-old. Several women also asked about why some babies cry “too much”, and what can be done.

Though not a primary focus of the planned content, the prenatal visit also became a forum for unanswered questions related to HIV care including maternal adherence to ART and HIV prophylaxis medication for infants as many mothers were unsure about how to administer the medication. The Lactation Specialist reinforced the importance of maternal adherence to ART and demonstrated how to measure out and administer medications to a newborn baby.

### Early postpartum needs

Around the time women gave birth, and between 2–4 weeks postpartum, our Research Coordinator received many phone calls from women with questions or concerns. Though it was not part of the intervention design that our Research Coordinator or Lactation Specialist would be available outside of the scheduled visits, participants reached out by phone at critical timepoints for support. For example, one woman phoned three days before the first postpartum visit because she felt she had no milk, her baby was crying too much, and neighbors were pressuring her to give her baby cow’s milk. In this case, the Research Coordinator was able to arrange for the initial postpartum session to occur earlier than planned and the Lactation Specialist supported the participant to achieve a more effective latch and position which allowed her baby to feed more effectively and for a longer period. Another woman reached out while still in the hospital because she was advised to wait several hours before initiating breastfeeding. In this case, the Research Coordinator was able to reassure the participant that she could initiate breastfeeding immediately after giving her baby the first dose of HIV prophylaxis medication as the Lactation Specialist had instructed. A number of women called saying their babies had “plastic teeth” and asking for advice. In these situations, the Lactation Specialist requested mothers bring their babies in to see her instead of taking their baby to a traditional healer for “plastic teeth” removal. Women heeded this advice and the Lactation Specialist was then able to examine the infants’ gums and reassure women there were no “plastic teeth”. Ultimately, by the time of the final session, no women had subjected their babies to “plastic teeth” removal.

### 2 weeks postpartum: 2nd breastfeeding support session

At around two weeks postpartum, women met with the Lactation Specialist and Research Coordinator again, this time with their babies in arm, and all women reported exclusively breastfeeding. After observing the participant breastfeeding, the Lactation Specialist assisted nearly all women to improve their breastfeeding technique including by helping women to adjust their baby’s latch and position to optimize comfort and effective milk exchange. Many women suffered from painful or cracked nipples, a result of poor latch. Several women also struggled with breast engorgement or babies preferring to nurse on only one side. The Lactation Specialist supported these women by assisting them to express milk from their engorged breast and by helping them find a comfortable latch and position for their baby on the non-preferred side. The Lactation Specialist also assisted one mother, whose baby was not able to maintain an effective latch, to express milk into a cup and feed her baby.

A majority of women were concerned that they may not have enough milk or that their babies were feeding too much, and many were already facing pressure from others to begin introducing complementary feeding. Thus, reviewing infant stomach sizes, signs of sufficient milk intake, the importance of feeding on demand and the benefits of breastfeeding exclusively for the first six months was important encouragement offered by the Lactation Specialist at this time. Despite concerns, based on her assessment and the infants’ weights, the Lactation Specialist did not identify any women with insufficient milk. For two babies, whose weight was less than expected at two weeks despite adequate milk (including the baby mentioned above who struggled to latch on), the Lactation Specialist made referrals to medical providers for further assessment, monitoring and care.

As women fed their babies during the session, the Lactation Specialist showed women the change in color and consistency between the foremilk and hindmilk by expressing a few drops of each onto a gloved hand. This was a powerful visual tool that surprised many women and showcased the two components of breast milk (hydration and nutrition). For some women, this demonstration facilitated continued exclusive breastfeeding because it was convincing evidence that providing water to breastfeeding babies was unnecessary.

In addition to the aforementioned breastfeeding concerns, a quarter of the women continued to have worries about “plastic teeth”. Many women also continued to worry about HIV transmission to their babies. In addition, most women sought support for at least one of a variety of other infant health concerns, the most common being rashes and concerns about crying or perceived colic pains. To address these concerns, the Lactation Specialist was able to examine the babies and reassure women that their babies had no “plastic teeth.” She was also able to advise about skin care, review the importance of burping babies to reduce gas pains, and make referrals to primary care doctors as needed. Finally, she eased worries about HIV transmission by reinforcing the importance of maternal and infant adherence and addressed follow-up or repeated questions about administering infant HIV prophylaxis medication.

At this time, some women also had questions about personal health concerns including infection of the cesarian section wounds, general exhaustion and fatigue. The Lactation Specialist advised on breastfeeding postures to reduce pressure on painful incisions and encouraged mothers to hydrate frequently and ask for help from supporters whenever possible. Women that needed additional medical attention were referred to relevant medical practitioners.

### 4-weeks postpartum: 3rd breastfeeding support session

The 4-week postpartum visit followed a similar flow to week 2 where the Lactation Specialist observed a feeding, gave feedback on technique, and addressed questions and concerns. All women still reported practicing exclusive breastfeeding at this time. The Lactation Specialist identified fewer issues with latch and positioning, but still made adjustments for some to improve technique. The infant who was unable to latch on at two-weeks was now able to latch on, but had not added as much weight as expected and fed hungrily from expressed milk given in a cup. The Lactation Specialist worked out a plan with this mother for her to give top-ups (around 120 milliliters of expressed breastmilk in a cup) after each breastfeeding.

At this time, many women still felt like their infants were feeding a lot and for some this led to continued worries about milk insufficiency. The Lactation Specialist took this opportunity to reassure women by reviewing the breastmilk supply and demand cycle, the reasons why infants cluster feed and infant growth and development. Women were also encouraged by seeing the increase in their infants’ weights over time. According to infant weight gain and the Lactation Specialist’s assessment, no women were suffering from insufficient milk supplies, though she had concern for two infants who had not gained weight as expected. One (described above), was to be given top-ups from the mother’s plentiful milk supply. The other infant, who had also been referred to medical providers at 2-weeks, returned at 4-weeks having gained very little weight and, on assessment, was not breastfeeding well. The Lactation Specialist was also concerned the mother looked detached. She encouraged and coached the mother supporting her to achieve an effective latch and comfortable position with skin-to-skin contact. She also encouraged her to offer top-ups of expressed breastmilk to her baby after each feeding. The Lactation Specialist then referred the baby for an early infant HIV test (normally done at six weeks), which was completed on the following day.

Compared to the two-week session, fewer women had concerns about HIV transmission to their babies or “plastic teeth”. Meanwhile, other questions and concerns were wide ranging including concerns about rashes, bowel patterns, colic, crying, the healing of umbilical cords, sleeping patterns, white colored tongues, oral thrush and a few remaining questions about infant HIV prophylaxis medications. The Lactation Specialist addressed women’s concerns by offering information, suggested interventions and referrals as appropriate. For example, she explained normal bowel patterns for breastfed babies, encouraged mothers not to overdress their babies (causing heat rash) and referred some for medications to treat thrush.

### 6-weeks postpartum: 4th breastfeeding session

At the six-week postpartum visit, all the women in our cohort still reported exclusively breastfeeding and the Lactation Specialist made very few adjustments to women’s position or latch. Of the two infants who struggled to breastfeed and gain weight at previous visits, both had gained weight as expected. One mother continued to express her milk after feeds and provide it in a cup to her baby. The Lactation Specialist worked with this mother to increase top-ups to continue to meet her growing baby’s needs. The other participant, whose baby was referred for early PCR testing, was still waiting on the test results, but showed improvements including a better latch, steady weight gain and more effective milk exchange.

Primary concerns of mothers at this time were cluster feeding (feeding too much/ too frequently) and perceived milk insufficiency. For women with these concerns, infant weights and assessments did not reveal milk insufficiency and the Lactation Specialist continued to provided reassurance and reviewed information about feeding patterns. In fact, the Lactation Specialist noted that a number of women were producing more than enough milk on one or both sides, and for some this led to engorgement. She assisted women to express milk, and made recommendations for warm compresses to relieve pain and in one case cold compresses to reduce milk production on one side. Only one infant had not gained weight as expected at this time. Upon assessment, the Lactation Specialist noted the mother still had foremilk after her baby had been feeding for around 30 min. The Lactation Specialist recommend the mother try expressing about 30 milliliters of foremilk before feeding her baby so that her baby would take in more hindmilk. She also recommended the mother apply gentle manual compression during feeding to stimulate increased milk ejection allowing her baby to take in more milk during each feed.

As with the 4-week visit, infant health was also a prominent concern for women at 6-weeks. Women asked about rashes, flu-like illnesses, infant’s tongue being white, gas/colic, thrush and baby’s bowel patterns—stool consistency, color and frequency and what to do when bowel patterns change. There were a small number of myths and cultural practices mentioned by women at this time including a persistent worry for some about “plastic teeth”. The Lactation Specialist continued to provide information, reassurance and referrals to women as needed. For example, she explained that a white tongue is often normal for a breastfeeding baby and requires no intervention.

### 3-months (12-weeks) postpartum: 5th infant feeding session

During this final session, all women reported exclusively breastfeeding. The Lactation Specialist assessed the health of women and their infants. When following up on participants with significant concerns the Lactation Specialist found the participant who had been primarily expressing milk and feeding her baby with a cup was now breastfeeding well. Her baby had gained more weight than expected, and she reported she had stopped giving top-ups at about eight weeks postpartum. The infant who was referred for early HIV testing at the 4-week visit had been diagnosed with HIV sometime after six weeks and promptly started on ART. The infant was now breastfeeding well and had gained weight since the last visit. The infant’s mother had also been found to have a very high viral load and was receiving additional support and counseling for her own adherence to ART. Finally, the baby of the mother with excess foremilk had gained weight as expected since the 6-week visit.

For a few women, worries around milk insufficiency persisted. These worries stemmed from babies being fussier and feeding more than usual. However, based on her assessment, satisfactory weight gain and the reported number of wet diapers, the Lactation Specialist was able to reassure women that their babies were adequately nourished. She reminded women that these were classic signs of a growth spurt, and that increased feeding was their baby’s way of stimulating an increase in milk supply.

At this 12-week visit, just a few women continued to worry about the possibility of transmitting HIV to their babies, but more than half had new or continued questions or concerns about their personal health or their baby’s general health. Several women had questions or concerns about when and how to access family planning.

Due to stockouts of reagents used for HIV testing, most women had their infant’s first HIV test (normally completed at six weeks postpartum) drawn at this time. This delay in infant testing underscores the importance of the referral made by the Lactation Specialist for early infant testing.

After addressing women’s concerns, the LS shared information to prepare women for complementary feeding (to be initiated after 6 months postpartum—refer to Table 4 for topics covered). The Lactation Specialist answered women’s questions and encouraged women to avoid a number of unadvisable, practices they brought up such as preparing the baby’s first porridge with up to seven cereals to (instead of the recommended two or three), giving babies powdered glucose, or “training” babies to eat by forced feeding. Finally, the Lactation Specialist addressed a number of misconceptions related to infant feeding such as the beliefs that allowing babies to sit up at this age would cause diarrhea, feeding babies eggs would interfere with their speech, or that a baby’s food should be boiled with water and salt only (no other spices and therefore must be prepared separately from the family’s meal). Much of the information presented on complementary feeding was also in the mother-child health booklet women received from the clinic. However, the booklet is published in English and many women had not read nor understood the booklet. Thus, this session was key to women’s understanding of current complementary feeding recommendations.

The session concluded with a demonstration of how to prepare the baby’s first foods, and many women were surprised by how thick the food should be and how little their babies would initially be consuming. Afterwards, a copy of the visual tool reminding women of the information discussed was given to women to take home.

## Discussion

Our findings reveal that the infant feeding support component of the *Supporting Healthy Mothers* trial was delivered largely as planned (feasible) with the content being relevant to women’s infant feeding experiences and high levels of attendance and retention (acceptable). Analysis of qualitative observations, clinical reports and phone call logs as well as in-depth discussions with the entire research team also point to the strengths and limitations of the personalized infant feeding support sessions, and highlighted areas for future development.

The Lactation Specialist delivered the planned curriculum and was also able to personalize the sessions addressing a number of specific questions and concerns outside the curriculum during the sessions. Good rapport with the Lactation Specialist likely prompted women to inquire about a broad range of topics, some unrelated to infant feeding. As such, several distinct topic areas emerged during the sessions that may be considered for further development including infant skin care, the safety of traditional practices and maternal self-care. Regular calls from participants with concerns, especially early postpartum, further points to the trust and confidence women had in the Research Coordinator and Lactation Specialist as well as the need women had for support on-demand during times of high vulnerability. Despite unexpected topics emerging and the number of phone calls fielded between sessions, the Lactation Specialist delivered the planned curriculum at the planned timepoints with no significant modifications.

We found the curriculum delivered aptly addressed women’s infant feeding challenges and concerns. Concerns regarding breastfeeding technique, milk insufficiency and the transmission of HIV through breastmilk as well as worries about “plastic teeth” decreased across time with support from the Lactation Specialist. The near perfect attendance by participants is noteworthy, especially considering several of the missed sessions occurred during the aftermath of Kenya’s presidential elections when civil unrest was feared. Furthermore, given the cash transfer was provided regardless of appointment attendance, the majority of women traveled 30 min or more to the clinic, and the frequency (every 2-weeks) of the early post-partum sessions, we believe this attendance record illustrates high acceptability of the intervention. Women benefited from the sessions and therefore willingly made an effort to attend even when they were not making a trip to the clinic for other patient visits.

We also found that holding *face-to-face* meetings with a skilled provider was important for identifying challenges that at times participants themselves had not identified. A myriad of remote or online interventions aimed at supporting perinatal women exist that are synergistic to in-person meetings [32–37]. However, we found that the Lactation Specialist’s physical assessments of women and their breastfeeding techniques led to key feedback including how to improve positioning and latch—important for effective milk exchange and milk supply development. Often the Lactation Specialist identified issues with technique the women themselves were not picking up on. Thus, without a face-to-face session, these issues would likely have gone undetected, potentially resulting in poor milk exchange, breast health issues and suboptimal infant growth. The Lactation Specialist also visually assessed many cases of engorged breasts and unequal breast sizes and offered suggestions for optimizing breast health and feeding accordingly. Furthermore, attending in-person sessions allowed women to appreciate the difference between foremilk and hindmilk, convincing many women that giving their babies water in addition to breastmilk (a common practice in this setting) [15] was unnecessary. In-person sessions also allowed us to collect infant weights. Infant weights have been used to assess the adequacy of early milk production in other settings, [38] and we found this was a key objective measure of breastmilk intake early postpartum. Typically, infant weights in this setting are only collected at birth and then again at six weeks postpartum. Thus, weights measured, on a highly accurate digital scale, at two weeks and four weeks postpartum provided important information to the Lactation Specialist and reassurance to mothers who worried about having enough milk. This was particularly important, given perceived milk insufficiency is widely known to be a primary reason for women to stop breastfeeding or prematurely introduce complementary foods [13, 14]. Finally, another important benefit of face-to-face sessions was allowing the Lactation Specialist to assess mother-infant bonding and identify any postpartum blues or depression concerns.

In addition to sessions being face to face, we found women needed regular support early postpartum to develop their breastfeeding technique, receive affirmation that their milk supply was adequate and ensure proper administration of their infant’s HIV prophylaxis medications. Early postpartum sessions allowed the Lactation Specialist to make timely referrals to medical providers for the few infants who were not thriving as expected. Interestingly, a common concern (especially at 2-weeks and 4-weeks postpartum) was the perception that infants were feeding “too much/ too frequently”. Given breastfeeding frequency early postpartum has been shown to directly impact the development of milk supply, [39] repeatedly reinforcing the milk supply/demand concept and the importance of feeding on demand was critical support for optimal breastfeeding early postpartum. Frequent, early postpartum support for breastfeeding mothers is also recommended in the WHO’s Infant and Young Child Feeding Counselling course manual which suggests, women and their infants should be assessed and supported at the time of birth, 2–4 days postpartum and 5–8 days postpartum, in addition to the timepoints our sessions were conducted [28]. The need for skilled support for breastfeeding initiation immediately postpartum has also been identified in other settings [40].

Frequent, personalized support for infant feeding through the early postpartum period was similarly part of the baby-friendly community initiative introduced in several counties in rural Kenya including, Kisumu County from 2014–2017 [41]. The baby-friendly community initiative offered 12 home-based counseling sessions—every two weeks from 37 weeks of pregnancy to one month after birth. The sessions were delivered in women’s homes by trained community health volunteers and effectively supported increased rates of exclusive breastfeeding [12]. Overall, there are unique benefits from support provided by clinic-based, skilled professionals as well as benefits to having lower cost more widely available community-based support. Thus, there is great potential for lay and professional healthcare workers to collaborate to support optimal infant feeding in future programming in this setting.

While frequent early postpartum visits from 2 weeks on and the accessibility of our team were key strengths of our lactation sessions, we identified areas for improvement. First of all, while we were able to address many early postpartum needs by fielding participant’s phone calls, support provided immediately postpartum could ensure infants properly receive their first dose of HIV prophylaxis and facilitate the prompt initiation of breastfeeding. Secondly, due to project time constraints, our last support session occurred when women were about three months postpartum. Three months postpartum was an ideal time for breastfeeding support since many babies go through a growth spurt at this time with bouts of cluster feeding. However, we would have ideally held another session closer to six months postpartum and provided women with the information about introducing the baby’s first foods and complementary feeding at that time. Other sessions beyond 6-months could also support women to continue breastfeeding beyond 12-months as most women planned to stop breastfeeding at around 12-months, well short of the recommended period (24-months).

### Limitations

Our assessment of feasibility does not include a cost benefit analysis which may be important when looking at ways to scale up this type of high-quality intensive support. Our assessment of acceptability includes limited direct input from participants in term of what they did or did not like and the perceived benefits of the support provided. Nevertheless, our detailed observations provide great insight as to how the infant feeding support was delivered and how it was received.

## Conclusions

The infant feeding support component of *Supporting Healthy Mothers* was successfully executed with high levels of attendance. The planned curriculum was feasible to deliver via face-to-face, personalized infant feeding support sessions from pregnancy to three months postpartum. Supportive sessions were highly acceptable to women who attended 94% of the sessions where their infant feeding concerns and challenges were addressed. At three months postpartum all women reported practicing exclusive breastfeeding as recommended. Qualitative data collected during the infant feeding support component of *Supporting Healthy Mothers* provide an overview of the breastfeeding support sessions including the primary concerns or challenges to optimal infant feeding women faced, how they were addressed by the Lactation Specialist at each session and areas where more support may be needed.

## Data Availability

The data on which this manuscript is based are not publicly available due to the need to protect the privacy of individual participants, but are available from the corresponding author on reasonable request.

## Declarations

### Authors ’ information (optional)

RS is the Lactation Specialist (LS) referred to throughout the manuscript. BCO is the Research Coordinator referred to throughout the manuscript.

## Abbreviations

HIV: Human Immunodeficiency Virus
WHO: World Health Organization
WLWH: Women Living with HIV
ART: Antiretroviral Therapy
PMTCT: Prevention of Mother to Child Transmission of HIV

## References

[1] Global Strategy for. Infant and young child feeding. World Health Organization; 2013.

[2] National Maternal, Infant and Young Child Nutrition Policy Guidelines Kenya. : Ministry of Health; Division of Nutrition; 2013.

[3] Guideline (2016). Updates on HIV and infant feeding: the duration of breastfeeding and support from health services to improve feeding practices among mothers living with HIV.

[4] UNAIDS FactSheet. 2021. UNAIDS; 2021.

[5] Pricilla RA, Brown M, Wexler C, Maloba M, Gautney BJ, Finocchario-Kessler S (2018). Progress toward eliminating mother to child transmission of HIV in Kenya: review of Treatment Guidelines Uptake and Pediatric Transmission between 2013 and 2016-A follow up. Matern Child Health J.

[6] Kenya AIDS, Response Progress Report. Nairobi, Kenya National Aids Control Council Health Mo; 2016.

[7] Guidelines on uses of Antiretroviral Drugs for Treating and Preventing HIV Infections in Kenya. Nairobi, Kenya Ministry of Health, National AIDS & STI Control Programme; 2016 Juy 2016.

[8] Kavle JA, LaCroix E, Dau H, Engmann C (2017). Addressing barriers to exclusive breast-feeding in low- and middle-income countries: a systematic review and programmatic implications. Public Health Nutr.

[9] Dunkley E, Ashaba S, Burns B, O'Neil K, Sanyu N, Akatukwasa C, et al. "I beg you...breastfeed the baby, things changed": infant feeding experiences among Ugandan mothers living with HIV in the context of evolving guidelines to prevent postnatal transmission. BMC Public Health. 2018;18(1):188. 10.1186/s12889-018-5081-x PMC5789624 29378548

[10] Odeny BM, Pfeiffer J, Farquhar C, Igonya EK, Gatuguta A, Kagwaini F (2016). The stigma of exclusive breastfeeding among both HIV-positive and HIV-negative women in Nairobi, Kenya. Breastfeed Med.

[11] Nabwera HM, Jepkosgei J, Muraya KW, Hassan AS, Molyneux CS, Ali R (2017). What influences feeding decisions for HIV-exposed infants in rural Kenya?. Int Breastfeed J.

[12] Samburu BM, Kimiywe J, Young SL, Wekesah FM, Wanjohi MN, Muriuki P (2021). Realities and challenges of breastfeeding policy in the context of HIV: a qualitative study on community perspectives on facilitators and barriers related to breastfeeding among HIV positive mothers in Baringo County, Kenya. Int Breastfeed J.

[13] Østergaard LR, Bula A (2010). They call our children nevirapine Bbabies? A qualitative study about exclusive breastfeeding among HIV positive mothers in Malawi. Afr J Reprod Health.

[14] Operto E (2020). Knowledge, attitudes, and practices regarding exclusive breastfeeding among HIV-positive mothers in Uganda: a qualitative study. Int J Health Plann Manage.

[15] Mbagaya GM. Child feeding practices in a rural western Kenya community. Afr J Prim Health Care Fam Med. 2009;1(1).

[16] Nabakwe EC, Egesah O, Kiverenge-Ettyang GA (2022). Maternal and health care workers’ perspectives on exclusive breastfeeding in the context of maternal HIV infection, in Busia county, western Kenya: a mixed methods cross-sectional survey. Int Breastfeed J.

[17] Mohamed MJ, Ochola S, Owino VO (2020). A qualitative exploration of the determinants of exclusive breastfeeding (EBF) Practices in Wajir County, Kenya. Int Breastfeed J.

[18] Tuthill EL, Maltby AE, Odhiambo BC, Akama E, Dawson-Rose C, Cohen CR et al. Financial and Food Insecurity are primary Challenges to Breastfeeding for Women living with HIV in Western Kenya: a longitudinal qualitative investigation. AIDS Behav. 2023. 10.1007/s10461-023-04046-8 PMC10577374 37043052

[19] Hazemba AN, Ncama BP, Sithole SL (2016). Promotion of exclusive breastfeeding among HIV-positive mothers: an exploratory qualitative study. Int Breastfeed J.

[20] Al-Mujtaba M, Sam-Agudu N, Khatri R (2016). Barriers to the practice of exclusive breastfeeding among HIV-positive mothers in sub-saharan Africa: a scoping review of counselling, socioeconomic and cultural factors. J AIDS HIV Res.

[21] Kinshella MW, Prasad S, Hiwa T, Vidler M, Nyondo-Mipando AL, Dube Q (2021). Barriers and facilitators for early and exclusive breastfeeding in health facilities in Sub-Saharan Africa: a systematic review. Glob Health Res Policy.

[22] Radzyminski S, Callister LC (2015). Health Professionals’ Attitudes and Beliefs about Breastfeeding. J Perinat Educ.

[23] Nyoni S, Sweet L, Clark J, Ward P (2019). A realist review of infant feeding counselling to increase exclusive breastfeeding by HIV-positive women in sub Saharan-Africa: what works for whom and in what contexts. BMC Public Health.

[24] Chabeda S, Oluoch D, Mwangome M, Jones C. Infant malnutrition treatment in Kenya: Health worker and breastfeeding peer supporter experiences. Matern Child Nutr. 2021;17(3). 10.1111/mcn.13148 PMC8189199 33528108

[25] Tuthill EL, Maltby AE, Odhiambo BC, Akama E, Pellowski JA, Cohen CR et al. I found out I was pregnant, and I started feeling stressed: a longitudinal qualitative perspective of mental health experiences among perinatal women living with HIV. AIDS Behav. 2021. 10.1007/s10461-021-03283-z PMC8126180 33997940

[26] Meleis AI. Afaf Meleis’ Transitions Theory. In: Smith M, editor. Nursing theories and nursing practice. 5th ed. F.A. Davis Company; 2019. pp. 353–70.

[27] Tuthill EL, Maltby AE, Odhiambo BC, Akama E, Dawson-Rose C, Weiser SD. Resilient mothering: an application of Transitions Theory from pregnancy to motherhood among women living with HIV in western Kenya. ANS Adv Nurs Sci. 2023. 10.1097/ANS.0000000000000478 PMC10354209 36656116

[28] WHO. Infant and young child feeding counselling: an integrated course. Trainer’s guide. Geneva World Health Organization; 2021.

[29] Maternal Infant and Young Child Nutrition. National Counseling Cards. In: Health Mo, editor.; 2010.

[30] Krueger R, Casey M (2009). Focus Groups: a practical guide for Applied Research.

[31] Mutai J, Muniu E, Sawe J, Hassanali J, Kibet P, Wanzala P (2010). Socio-cuItural practices of deciduous canine tooth bud removal among maasai children. Int Dent J.

[32] Morse H, Brown A (2021). Accessing local support online: mothers’ experiences of local Breastfeeding Support Facebook groups. Matern Child Nutr.

[33] Zunza M, Cotton MF, Mbuagbaw L, Lester R, Thabane L (2017). Interactive weekly mobile phone text messaging plus motivational interviewing in promotion of breastfeeding among women living with HIV in South Africa: study protocol for a randomized controlled trial. Trials.

[34] Awiti PO, Grotta A, van der Kop M, Dusabe J, Thorson A, Mwangi J (2016). The effect of an interactive weekly mobile phone messaging on retention in prevention of mother to child transmission (PMTCT) of HIV program: study protocol for a randomized controlled trial (WELTEL PMTCT). BMC Med Inform Decis Mak.

[35] Seguranyes G, Costa D, Fuentelsaz-Gallego C, Beneit JV, Carabantes D, Gomez-Moreno C (2014). Efficacy of a videoconferencing intervention compared with standard postnatal care at primary care health centres in Catalonia. Midwifery.

[36] Unger JA, Ronen K, Perrier T, DeRenzi B, Slyker J, Drake AL (2018). Short message service communication improves exclusive breastfeeding and early postpartum contraception in a low- to middle-income country setting: a randomised trial. BJOG.

[37] Mangwi Ayiasi R, Kolsteren P, Batwala V, Criel B, Orach CG (2016). Effect of Village Health Team Home visits and mobile phone consultations on maternal and Newborn Care Practices in Masindi and Kiryandongo, Uganda: a community-intervention trial. PLoS ONE.

[38] Kent JC, Gardner H, Geddes DT. Breastmilk Production in the First 4 weeks after birth of term infants. Nutrients. 2016;8(12). 10.3390/nu8120756 PMC5188411 27897979

[39] Huang SK, Chih MH (2020). Increased breastfeeding frequency enhances milk production and infant weight gain: correlation with the basal maternal prolactin level. Breastfeed Med.

[40] Kervin BE, Kemp L, Pulver LJ (2010). Types and timing of breastfeeding support and its impact on mothers’ behaviours. J Paediatr Child Health.

[41] Kavle JA, Ahoya B, Kiige L, Mwando R, Olwenyi F, Straubinger S (2019). Baby-Friendly Community Initiative-From national guidelines to implementation: a multisectoral platform for improving infant and young child feeding practices and integrated health services. Matern Child Nutr.

